# Dosimetric Planning Comparison for Left-Sided Breast Cancer Radiotherapy: The Clinical Feasibility of Four-Dimensional-Computed Tomography-Based Treatment Planning Optimization

**DOI:** 10.7759/cureus.24777

**Published:** 2022-05-06

**Authors:** Oi-Wai Chau, Hatim Fakir, Michael Lock, Robert Dinniwell, Francisco Perera, Abigail Erickson, Stewart Gaede

**Affiliations:** 1 Physics and Engineering, London Regional Cancer Program, London, CAN; 2 Radiation Oncology, Schulich School of Medicine and Dentistry, Western University, London, CAN; 3 Department of Radiation Oncology, Western University, London, CAN; 4 Radiation Oncology, London Regional Cancer Program, Western University, London, CAN; 5 Division of Radiation Oncology, London Regional Cancer Program, London, CAN; 6 Physics and Engineering, Department of Medical Physics, London Regional Cancer Program, London, CAN

**Keywords:** distal left anterior descending artery (lad), left ventricle, deep-inspiration breath-hold, 4d-ct, dosimetric planning, cardiac, radiotherapy, left-sided breast cancer

## Abstract

Background: Adjuvant whole-breast radiotherapy (RT) is a significant part of the standard of care treatment after breast cancer (BC) conserving surgery. Modern techniques including intensity-modulated radiation therapy (IMRT) and volumetric-modulated arc therapy (VMAT) have constituted to better target coverage and critical organs sparing. However, BC survivors are at risk of developing radiation-induced cardiac toxicity. Hence, deep-inspiration breath-hold (DIBH) techniques have been implemented at many centers to further reduce cardiac exposure but require compliance. 4D-CT robust optimization can account for heart intrafractional motion per breathing phase. The optimization has been explored in cardiac sparing of breast IMRT compared to DIBH in a small sample size but has not been evaluated in substructures sparing, nor in VMAT. To provide patients who are not compliant to breath-hold with an optimal treatment approach, various heart sparing techniques need to be evaluated for statistical significance and clinical feasibility.

Aim: This retrospective study aimed to provide an extensive dosimetric heart sparing comparison of free-breathing, 4D-CT-based treatment planning, including robust optimization with DIBH-based treatment planning. Combinations of forward and inverse IMRT and VMAT are also considered.

Methods: Fifteen early stage left-sided BC standard treatment plans were selected. Breast, lung, left anterior descending artery (LAD), left ventricle (LV), and the whole heart were contoured on each 4D-CT phase and DIBH CT dataset. Each treatment plan was optimized using forward/inverse IMRT and VMAT on the following CT datasets: DIBH, average 4D-CT, and the complete 4D-CT dataset needed for robust optimization. Dose-volume histograms were used to compare V_5Gy_Heart, mean heart dose, mean and max LAD dose, mean LV dose, and V_50%_Lung.

Results: All RT techniques assessed including 4D robust optimization were clinically feasible. Statistically significant differences in mean heart, LAD and LV dose, max LAD dose, and V_5Gy_Heart (p < 0.01) but no difference in V_50%_Lung (p = 0.29) were found between different techniques. IMRT DIBH achieved the optimal cardiac and substructure sparing among treatment plans. 4D robust IMRT had significantly greater mean heart and LV dose than DIBH IMRT (p ≤ 0.01), except LAD dose. Among free-breathing methods, no difference in all cardiac and substructure dose parameters was observed (p > 0.2) in comparing forward and inverse IMRT with average 4D-CT, inverse average 4D-CT, and 4D robust with IMRT, and between average 4D-CT VMAT and 4D robust VMAT. Only V_5Gy_Heart and mean LV dose were significantly greater in 4D robust VMAT (p < 0.01) compared to DIBH VMAT. Mean heart and LV doses were significantly reduced (p < 0.01) in DIBH IMRT compared to DIBH VMAT. Moreover, mean heart and LV dose, V_5Gy_Heart were significantly reduced in inverse IMRT average 4D-CT compared to average 4D-CT VMAT (p < 0.02) and in 4D robust IMRT compared to 4D robust VMAT (p < 0.04).

Conclusion: This study demonstrated the clinical feasibility of 4D robust optimization in limiting the cardiac and substructures dose during free-breathing RT with both IMRT/VMAT for patients who are not compliant with breath-hold RT. However, this study also presents that 4D robust optimization can reduce LAD dose but not fully outperform DIBH or conventional 4D-CT-based planning with IMRT/VMAT in heart sparing in treating early staged left-sided BC patients.

## Introduction

Breast cancer (BC) is the most common cancer in women worldwide. Adjuvant radiation therapy (RT) is commonly used after breast-conserving surgery to increase overall survival by decreasing the rate of cancer recurrence [1]. Traditionally, 3D conformal RT with tangential field directions is used in breast RT [2]. More modern techniques, including intensity-modulated radiation therapy (IMRT) and volumetric-modulated arc therapy (VMAT), have been shown to provide higher target coverage and more effective sparing of critical organs [3-5]. However, a proportion of BC survivors develop the radiation-induced cardiac disease later in their cure life [6]. Darby et al. concluded a linear relationship between major coronary events such as myocardial infarction and death from ischemic heart disease and radiation dose without a threshold (7.4% per Gy of mean heart dose) [7].

Traditionally, mean heart dose is commonly used as a reference measure for treatment planning constraints and in cardiotoxicity studies. However, there is increased evidence that cardiac substructure dose is associated with radiation-induced heart disease. Nilsson et al. previously reported an increase of stenosis in the left anterior descending artery (LAD) in irradiated left-sided BC patients and an association between high-risk RT and stenosis in hotspot areas for radiation, which indicated a linkage between radiation and location of coronary stenosis [8]. van den Bogaard et al. performed a group dose-distribution analysis and showed that the left ventricle (LV) received the highest dose among all cardiac structures due to the LV position relative to the left breast [2]. Arslan et al. retrospectively evaluated LV and LAD dose sparing in patients treated with free-breathing left-sided breast IMRT delivered with an additional boost, presented with significant reductions in the mean and max dose of the cardiac substructures in the re-optimized plan [9]. Currently cardiac and its substructure dosimetric consensus constraints have not been fully evaluated nor established. Beaton et al. performed a retrospective case-control-matched study and found that the risk of radiation-induced cardiac death at 10 years appears to be very low if mean heart dose is <3.3 Gy and maximum LAD dose (EQD2_3_ Gy) is <45.4 Gy [10]. Furthermore, cardiac dose parameters are limited and vary in endpoints. Based on the Quantitative Analyses of Normal Tissue Effects in the Clinic (QUANTEC) guidelines stated, the V_25Gy_Heart to be <10% to decrease the cardiac mortality to <1% [11]. Hence, it is important to evaluate not just the radiation dose to the heart, but also the radiation to which the substructures are subjected.

Deep-inspiration breath-hold (DIBH) has been implemented in the BC RT routine to further reduce cardiac exposure to irradiation in many centers [12]. The heart moves posteriorly and inferiorly during deep inspiration due to lung expansion and diaphragmatic movements. This maximizes the distance between the breast and the heart and reduces cardiac dose deposition. Dose planning and clinical studies have concluded that moderate DIBH is efficient and can effectively decrease mean heart dose in BC patients [13-15]. While DIBH techniques can potentially lower the radiation dose to the heart, it still requires patient compliance and longer treatment duration.

The RayStation treatment planning system v7 (RaySearch Laboratories, Sweden) optimization technique run in a Graphics Processor Unit (GPU) system and can account for heart intrafractional motion at each breathing phase, if a 4D-CT scan is acquired. This method is called 4D robust optimization. It has been used in RT to account for position uncertainties including patient setup and tumor motion relative to the target volume during treatment delivery [16-17]. 4D robust optimization utilizes min-max optimization to ensure dose planning stability by maximizing the plan quality in the worst-case scenario [18]. In contrast to conventional untagged average 4D-CT treatment planning, in which the internal target volume is expanded with a fixed margin to create the planning target volume (PTV) that encompasses the end-inhale and end-exhale target volume, robust optimization discretizes each phase of 4D-CT target volumes into multiple scenarios. The min-max optimization method allows the prescription to hold true even in the worst-case scenario. And performing the dose calculation with the optimized treatment plan on the end-inhale 4D-CT dataset instead of the average 4D-CT dataset. The additional respiration motion information obtained from 4D-CT is useful. El-Sherif et al. showed that dose estimates for the LAD were substantially susceptible to intrafraction respiratory motion, adjunct to small ranges of dose to the heart and LV [19]. The robust optimization technique has been explored by Mahmoudzadeh et al. in the context of cardiac sparing for breast IMRT in a limited sample size under normal free-breathing conditions (six patients) and controlled breath-hold conditions using the active breathing control (two patients) [20]. However, like Darby et al., this study focused on whole heart dosimetric parameters only and did not take into account the dose to cardiac substructures, such as the LAD and the LV [7]. Direct comparison among techniques of 4D robust optimization, DIBH, and standard 4D in combination with forward and inverse IMRT or VMAT has not been done in the literature.

In this study, we performed an extensive dosimetric comparison among various treatment planning techniques. Fixed-beam tangents, IMRT, and VMAT along with motion management strategies, including traditional motion encompassment with 4D-CT, 4D-CT robust optimization, and DIBH. This work aims to: (1) identify the clinical feasibility of the aforementioned techniques in sparing the heart and its substructures; (2) address whether 4D robust optimization can outperform DIBH and conventional 4D-CT techniques; and (3) determine the clinical feasibility of IMRT/VMAT versus conventional tangents.

This article was previously presented as a poster meeting abstract at the 2020 American Association of Physicists in Medicine (AAPM) Annual Scientific Meeting on July 12, 2020.

## Materials and methods

Patient selection

Fifteen consecutive early stage (T0-T2A) left-sided BC patients who were treated from 2018 to 2019 with standard breast RT plans were selected. Mean age of patients was 60 ± 12 years (range 35-76).

CT simulation and delineation

Both DIBH and free-breathing four-dimensional computed tomography (4D-CT) simulations were performed on each patient using the Philips Brilliance Big Bore CT scanner (Philips Medical Systems, Cleveland, USA). Simulation imaging was performed with the patient in supine position, with scans taken from the upper border of the hyoid bone to the diaphragm. 4D-CT scans were reconstructed into 10 phases using the Respiratory Gating for Scanners (RGSC) system (Varian Medical Systems, Palo Alto, USA). An untagged average 4D-CT dataset (UNTAG AVERAGE) that uses all the projection data acquired during the low-pitch helical CT scan was also generated and served as the primary dataset for the free-breathing scenario.

The breast volume was contoured, according to the delineation guidelines for adjuvant radiotherapy (RT) of early BC by the Radiation Therapy Oncology Group (RTOG) and was approved by breast oncologists [21]. The anterior component of the target volume contours of the left breast was defined by a 5 mm contraction of the external contour. The pericardium was defined as the border of the LAD and the whole heart, while the LV was contoured from the top LV border to the apex cordis. The LAD, LV, both lungs, spinal cord, and the whole heart were delineated on all 10 phases of the 4D-CT dataset, the untag average dataset, and the DIBH dataset (with supervision of a radiation oncologist) using RayStation 7 software (RaySearch Laboratories, Sweden).

Treatment planning

Eight treatment plans were generated for each patient. Forward IMRT, inverse IMRT, and VMAT were optimized on both the DIBH and UNTAG AVERAGE datasets. Additionally, 4D robust IMRT and VMAT optimization, which uses the entire 4D-CT dataset, were also performed, giving a total of 120 treatment plans. A dose of 4250 cGy in 16 fractions, the current clinical standard in our clinic, was prescribed for each patient, with a minimum of 90% coverage of the left breast target volume. The dose was calculated using collapsed cone convolution.

VMAT planning was composed of 2-4 partial arc beams ranging from 300° to 180° in the clockwise and counter-clockwise directions. Dose optimization for fixed tangential beams was performed using both forward and inverse step-and-shoot treatment planning techniques. The beam energy was chosen based on the distance between the radiopaque markers on the anterior-posterior set-point: 6 MV for separations less than 20 cm, 10 MV for separations between 20 and 23 cm, and 15 MV (with or without additional 6 MV beams) for separations larger than 23 cm.

Objectives and constraints goals were prioritized as follows: (1) heart; (2) left lung; (3) left breast; (4) right breast; (5) right lung; and (6) remaining normal tissue, according to QUANTEC and dosimetric guidelines in our clinic (Table 1) [9]. For the LV and LAD, no constraint was set up due to the lack of literature consensus guidelines available. However, the doses of these two substructures were aimed to be reduced as low as possible.

**Table 1:** Objectives and constraints goals for inverse-IMRT and VMAT treatment plans with and without robust optimization DVH: dose-volume histogram; EUD: equivalent uniform dose; IMRT: intensity-modulated radiation therapy; VMAT: volumetric-modulated arc therapy

Objectives	Min dose: Left breast target volume with 4250 cGy
	Max DVH: 1% of left breast target volume with 4460 cGy; 10% of heart with 250 cGy; 8% of heart with 1000 cGy; 30% of left lung with 250 cGy; 10% of left lung with 1200 cGy
	Dose fall-off: Left breast target volume: 4200 cGy to 4000 cGy in 2 mm
	Max EUD: Spinal cord: 17 cGy; right breast: 240 cGy; right lung: 37 cGy
	Uniform dose: Left breast target volume: 4250 cGy
Constraints	Min DVH: 97% of left breast target volume with 4250 cGy
	Max Dose: Left breast target volume: 4460 cGy

Dosimetric assessment

Dose-volume histograms (DVHs) were used to compare the following parameters: V_5Gy_Heart (the volume of heart receiving at least 5 Gy, V_50%_Lung; total lung volume receiving at least 2125 cGy) and the mean heart, mean LAD, mean LV dose, and max LAD dose. Statistical analyses were performed in SPSS IBM v.23 (IBM SPSS Statistics for Windows, Armonk, NY) using the Shapiro-Wilk test for normality, Kruskal-Wallis nonparametric one-way analysis of variance (ANOVA) to test for significance, and Wilcoxon-Mann-Whitney test to find between-subject significance.

## Results

**Figure 1:**
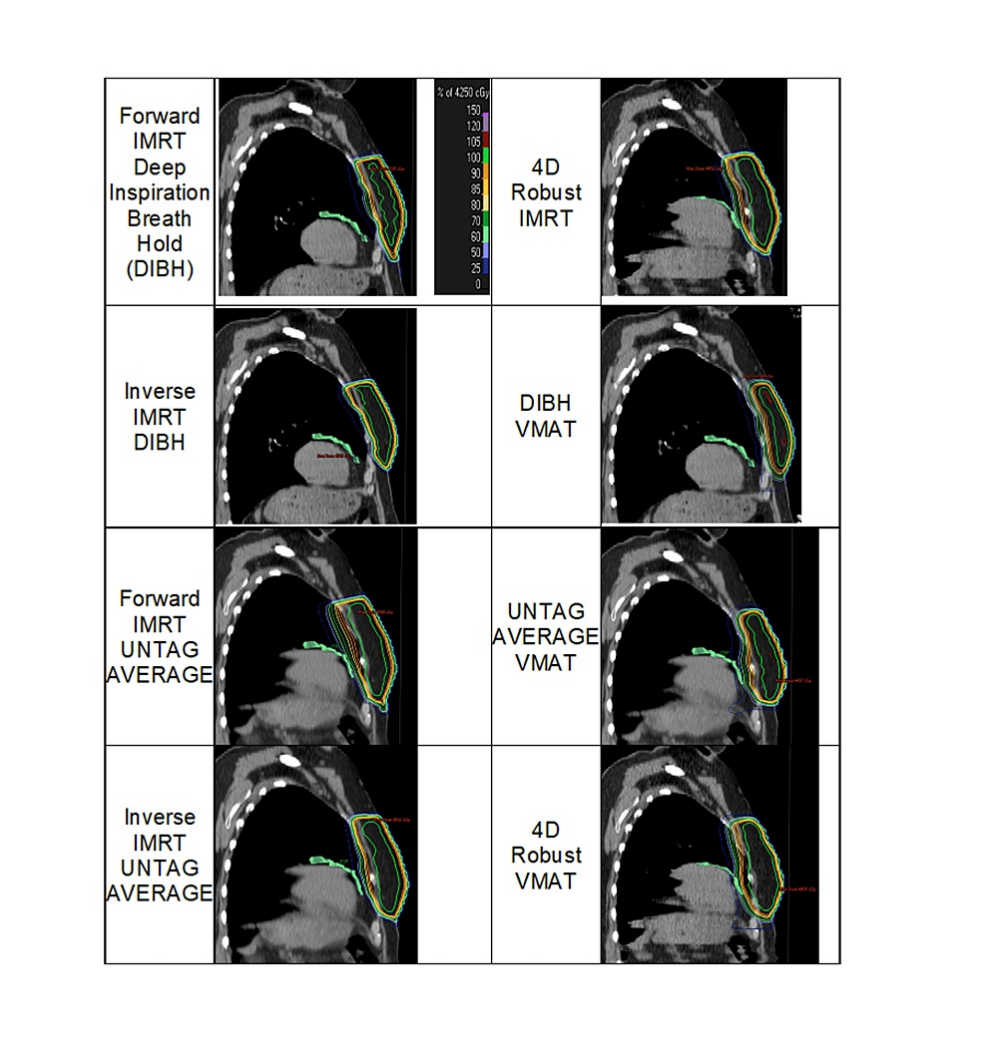
Whole-breast radiation treatment methods, with corresponding dose distribution of a representative left-sided breast cancer patient. The left anterior descending artery was three-dimensionally segmented in green on each CT image. Noted that the 4D robust and UNTAG AVERAGE radiation treatments were planned on the same 4D-CT dataset, for simplicity, the dose distributions of 4D robust technique were overlaid on the end-inspiration CT image of the 4D-CT dataset. IMRT: intensity-modulated radiation therapy; DIBH: deep-inspiration breath-hold; UNTAG AVERAGE: Untagged average 4D-CT dataset; 4D robust: 4D-CT robust optimization dataset

All treatment plans generated from the eight RT planning techniques were clinically feasible and reviewed by a certified dosimetrist, with a selection of sample patients contours approved by the radiation oncologists. All achieved a minimum of 90% coverage of 4250 cGy prescription dose in 16 fractions. Dose distributions for each of the eight planning techniques for a representative patient are displayed in Figure 1.

All cardiac and substructure dose metrics obtained from the eight planning techniques fulfilled the literature recommended constraints [11]. This included a mean heart dose below 330 cGy which was reported by Saiki et al. in BC patients with outcomes of congestive heart failure, and below 280 cGy which was corresponded to a significant elevated risk in coronary heart disease from a peptic ulcer disease study [22,23]. Furthermore, all methods achieved a V_5Gy_Heart <12%, mean LV dose <670 cGy, mean LAD dose <2380 cGy, and a max LAD dose <4780 cGy, which were reported by Skyttä et al. in patients who received breast RT with an outcome of >30% increase in cardiac damage biomarker, serum troponin T (hscTNT) [24]. The mean LAD dose was below 1000 cGy, which was the suggested constraint from the Expert Panel of the German Society of Radiation Oncology (DEGRO) [25]. And the max LAD dose was below 4540 cGy, which corresponded to a lower risk of radiation-induced cardiac death presented by Beaton et al. [10].

All dose parameters apart from the mean heart dose were not normally distributed (p < 0.05) in each of the eight treatment methods, especially for inverse IMRT DIBH. Table 2 displays the mean values for each parameter from the eight treatment planning techniques and the corresponding p-values from Kruskal-Wallis one-way ANOVA test. Only V_50%_Lung (p = 0.29) was statistically equal among planning techniques.

**Table 2:** Mean values +/- standard deviation of all parameters compared. A p-value < 0.05 determines significance from Kruskal-Wallis one-way analysis of variance. IMRT: intensity-modulated radiation therapy; DIBH: deep-inspiration breath-hold; UNTAG AVERAGE: untagged average 4D-CT dataset; 4D robust: 4D-CT robust optimization dataset; V5GyHeart: volume of heart receiving at least 5 Gy; V50%Lung: total lung volume receiving at least 2125 cGy

	Mean heart dose (cGy)	V_5Gy_Heart (%)	Mean LV dose (cGy)	Mean LAD dose (cGy)	Max LAD dose (cGy)	V_50%_Lung (%)
Forward IMRT DIBH	74 ± 27	1.12 ± 1.43	89 ± 29	231 ± 141	976 ± 744	3.04 ± 1.54
Inverse IMRT DIBH	70 ± 30	1.37 ± 1.55	82 ± 34	242 ± 175	1112 ± 842	2.61 ± 1.29
Forward IMRT UNTAG AVERAGE	148 ± 58	4.06 ± 2.25	192 ± 68	453 ± 229	2351 ± 1057	3.44 ± 1.59
Inverse IMRT UNTAG AVERAGE	121 ± 38	3.43 ± 1.84	172 ± 6	379 ± 265	1987 ± 1268	2.4 ± 1.2
4D robust IMRT	120 ± 52	2.69 ± 1.82	170 ± 74	258 ± 120	1444 ± 959	2.28 ± 1.62
DIBH VMAT	147 ± 13	1.75 ± 1.7	176 ± 25	296 ± 123	1059 ± 743	3.22 ± 0.6
UNTAG AVERAGE VMAT	188 ± 36	5.39 ± 2.04	246 ± 56	370 ± 173	1530 ± 753	2.97 ± 1.25
4D robust VMAT	173 ± 45	4.42 ± 2.56	245 ± 82	299 ± 101	1220 ± 716	3.13 ± 1.16
p-value	<0.0001	<0.0001	<0.0001	0.008	0.002	0.287

Results from the Wilcoxon-Mann-Whitney test showed that only forward and inverse IMRT DIBH techniques were considered equal for all dose parameters (p > 0.05) (Table 3).

**Table 3:** P-values of each parameter obtained from Wilcoxon-Mann-Whitney test comparing each planning method to forward IMRT DIBH technique. A p-value < 0.05 determines significant difference compared to forward IMRT DIBH technique. IMRT: intensity-modulated radiation therapy; DIBH: deep-inspiration breath-hold; UNTAG AVERAGE: untagged average 4D-CT dataset; 4D robust: 4D-CT robust optimization dataset; V5GyHeart: volume of heart receiving at least 5 Gy; V50%Lung: total lung volume receiving at least 2125 cGy

	Planning method	Inverse IMRT DIBH	Forward IMRT UNTAG AVERAGE	Inverse IMRT UNTAG AVERAGE	4D robust IMRT	DIBH VMAT	UNTAG AVERAGE VMAT	4D robust VMAT
P-value	Mean heart dose (cGy)	0.62	0.00	0.00	0.00	0.00	0.00	0.00
	V_5Gy_Heart (%)	0.68	0.00	0.00	0.01	0.19	0.00	0.00
	Mean LV dose (cGy)	0.33	0.00	0.00	0.00	0.00	0.00	0.00
	Mean LAD dose (cGy)	0.84	0.00	0.07	0.41	0.06	0.01	0.02
	Max LAD dose (cGy)	0.57	0.00	0.02	0.16	0.84	0.02	0.27
	V_50%_Lung (%)	0.41	0.51	0.25	0.22	0.62	0.97	0.78

**Figure 2:**
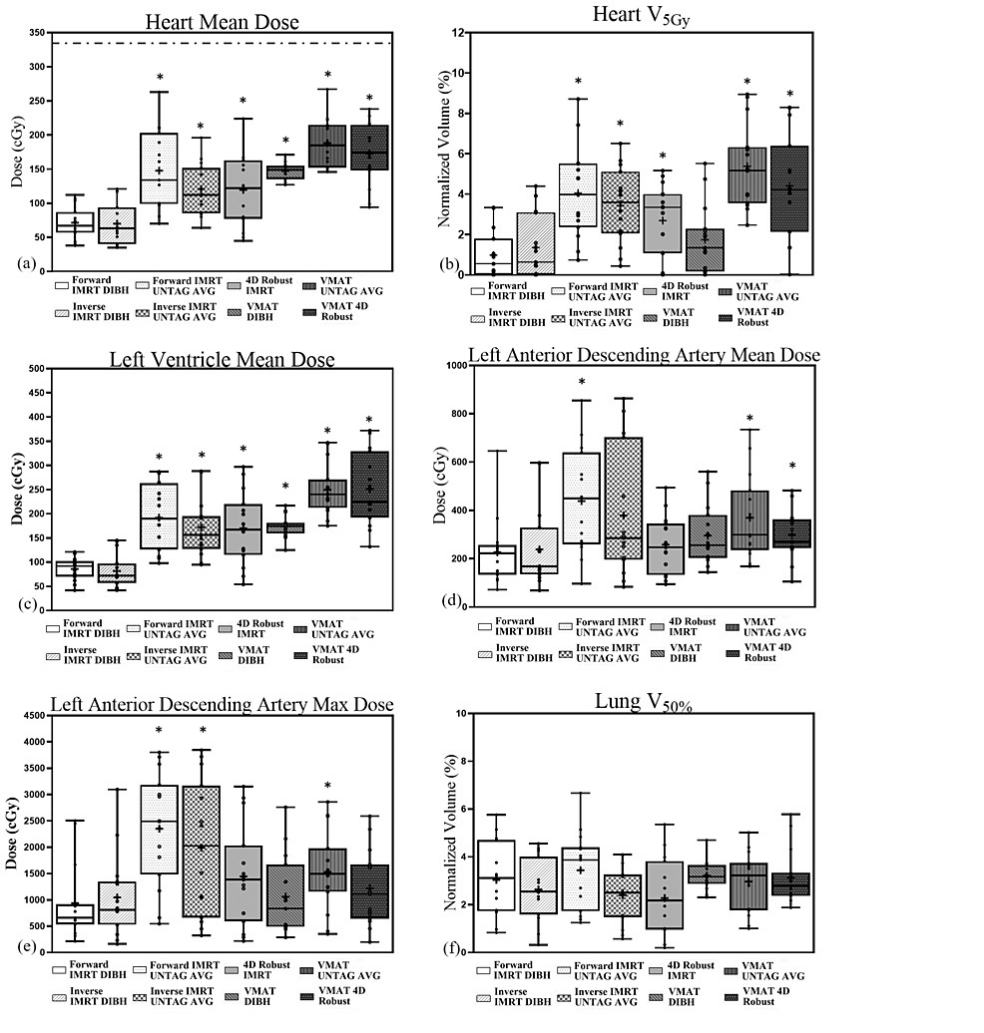
Boxplot displaying (a) mean heart dose, under literature threshold of 330 cGy, (b) V5GyHeart, (c) mean left ventricle dose, (d) mean left anterior descending artery dose, (e) max left anterior descending artery dose, (f) V50%Lung. The plots whiskers displayed the minimum and the maximum value, the mean value was indicated with “+”. Wilcoxon-Mann-Whitney test results of significant difference between planning methods compared to forward IMRT DIBH technique was indicated with “*”. IMRT: intensity-modulated radiation therapy; DIBH: deep-inspiration breath-hold; UNTAG AVG: untagged average 4D-CT dataset; 4D robust: 4D-CT robust optimization dataset; V5GyHeart: volume of heart receiving at least 5 Gy; V50%Lung: total lung volume receiving at least 2125 cGy

In Figures 2a-2f, a significant difference between parameters from each planning method compared to forward IMRT DIBH was indicated with *.

Table 4 shows the p-value results from the Wilcoxon-Mann-Whitney test comparing each parameter between IMRT and VMAT and between 4D robust optimization, DIBH, and standard 4D UNTAG AVERAGE treatment plans.

**Table 4:** The p-value results from Wilcoxon-Mann-Whitney test comparing each parameter between free-breathing IMRT and VMAT and among 4D robust, DIBH, and standard 4D UNTAG AVERAGE treatment plans. IMRT: intensity-modulated radiation therapy; DIBH: deep-inspiration breath-hold; UNTAG AVG: untagged average 4D-CT dataset; 4D robust: 4D-CT robust optimization dataset; V5GyHeart: volume of heart receiving at least 5 Gy

	Planning method	Forward vs inverse IMRT UNTAG AVG	Inverse IMRT UNTAG AVG vs 4D robust IMRT	Inverse IMRT UNTAG AVG vs UNTAG AVERAGE VMAT	4D robust IMRT vs 4D robust VMAT	UNTAG AVG VMAT vs 4D robust VMAT	DIBH VMAT vs 4D robust VMAT
P-value	Mean heart dose (cGy)	0.237	0.934	<0.001	0.011	0.561	0.051
	V_5Gy_Heart (%)	0.548	0.281	0.026	0.034	0.395	0.004
	Mean LV Dose (cGy)	0.547	0.95	0.002	0.007	0.852	0.001
	Mean LAD dose (cGy)	0.431	0.407	0.724	0.272	0.351	0.724
	Max LAD dose (cGy)	0.419	0.254	0.443	0.604	0.272	0.419

In correspondence to objective (2) to address whether 4D robust optimization can outperform DIBH and standard 4D, Figures 2a-2f showed that 4D robust IMRT had statistically significantly greater V_5Gy_Heart, mean heart, and LV dose compared to DIBH IMRT (p ≤ 0.01), except for mean and max LAD dose (p > 0.1). In comparison, DIBH with forward IMRT achieved a significant reduction in mean heart dose, V_5Gy_Heart, mean LV, and max LAD dose compared to free-breathing UNTAG AVERAGE IMRT (p ≤ 0.02) except for mean LAD dose (p = 0.07). In Table 4, among free-breathing methods, no difference in all cardiac and substructure dose including LAD and LV parameters were found (p > 0.2) in comparing between forward and inverse IMRT UNTAG AVERAGE, between inverse IMRT UNTAG AVERAGE and 4D robust IMRT, and between UNTAG AVERAGE VMAT and 4D robust VMAT. Among VMAT techniques, V_5Gy_Heart and mean LV dose were significantly reduced in DIBH (p < 0.005) compared to 4D robust VMAT, mean heart and LAD dose, max LAD dose were not different.

In correspondence to objective (3) to address the clinical feasibility, to determine whether we can reduce the complexity by using IMRT instead of VMAT. From Figures 2a-2f, DIBH IMRT had significantly less mean heart and LV dose (p < 0.01) than DIBH VMAT, whereas V_5Gy_Heart, mean LAD, and max LAD dose were not different (p ≥ 0.05). And from Table 4, inverse IMRT UNTAG AVERAGE had significantly less mean heart and LV dose, V_5Gy_Heart compared to UNTAG AVERAGE VMAT (p < 0.02), mean and max LAD dose were not different (p > 0.4). Furthermore, 4D robust IMRT had significantly less mean heart and LV dose, V_5Gy_Heart compared to 4D robust VMAT (p < 0.04), mean and max LAD dose were not different (p > 0.2).

## Discussion

In BC patients, it is recommended to minimize the irradiated cardiac volume without compromising the target coverage to reduce the risk of radiation-induced cardiac toxicity (RICT) without additional risk of recurrence in their later life post-RT. Radiation-induced cardiac effects in the early stage can lead to microvascular injuries caused by irradiating the myocardial endothelial cells, which can lead to acute inflammatory response, vascular damage, and fibrosis [26]. Macrovascular damage can be seen as a latent effect, where the atherosclerotic process accelerates in the coronary arteries. Therefore, it is important to minimize the radiation toxicity with the use of sparing techniques, which target the heart and its substructures from radiation.

This comprehensive treatment planning study investigated cardiac sparing techniques including respiratory motion management techniques such as 4D-CT encompassment and DIBH, as well as advanced RT techniques such as forward/inverse IMRT and VMAT. The scope of treatment planning techniques evaluated in this study has not been comprehensively performed in the literature. The heart and its substructure doses including the mean dose to the LAD and LV and max dose to LAD were evaluated in each plan. We were able to achieve clinically acceptable plans for all eight techniques including 4D robust optimization according to current guidelines from QUANTEC and the literature [10,11,22-25]. Thus, we have shown the capability and clinical feasibility of free-breathing 4D-CT-based RT for patients who are not compliant with breath-hold RT and for centers where DIBH is limited.

In this study, 4D robust IMRT had significantly greater cardiac and LV doses compared to DIBH IMRT (p ≤ 0.01), but mean and max LAD doses were not different (p > 0.1). Furthermore, no significant difference between free-breathing IMRT methods (UNTAG AVERAGE IMRT and 4D robust IMRT) was found. The mean LAD dose was not different comparing inverse UNTAG AVERAGE IMRT and DIBH IMRT (p ≥ 0.07). This proved that the use of 4D robust optimization was clinically feasible and provided a further limitation of radiation dose to the LAD, but not to the heart and LV during free-breathing IMRT treatment for patients who are not compliant with breath-hold (compared to standard 4D-CT-based RT). In the literature, tangential treatment planning reported a mean heart dose and/or mean LV dose reduction using DIBH tangential RT in comparison to free-breathing tangential RT [13,27-29]. Note, however, that these studies did not consider 4D-CT data sets and 4D robust optimization. In contrast, our results showed that DIBH IMRT can significantly reduce the heart and LV dose but not LAD dose compared to free-breathing techniques. These results agreed with Mahmoudzadeh et al., who showed that 4D robust optimization can potentially reduce, but not fully replace, the need for breath-hold in the tangential IMRT and can be applied to any case treated under free‐breathing [20].

Insignificant differences in all dose parameters (p > 0.05) between UNTAG AVERAGE VMAT and 4D robust VMAT, along with significant reduction in mean LV dose and V_5Gy_Heart (p < 0.01) in DIBH VMAT compared to UNTAG AVERAGE VMAT were found in our study. However, mean heart dose, LAD dose, and max LAD dose were not different compared to DIBH VMAT and 4D robust VMAT. This is contrary to the findings reported by Sakka et al., in which mean heart and LAD doses in DIBH VMAT were significantly reduced compared to free-breathing VMAT [28]. Therefore, the use of 4D robust optimization provided further radiation dose reduction to the LAD, but not the heart nor the LV compared to DIBH and standard 4D-CT-based VMAT.

Significant reduction in mean heart and mean LV dose was observed in DIBH tangential IMRT compared to DIBH VMAT in our study (p < 0.01). This result was aligned with literature findings [4,29]. Significant reduction in mean heart and LV dose and V_5Gy_Heart was observed in 4D-CT-based-IMRT compared to 4D-CT-based VMAT (p < 0.04). This was supported by Sakka et al. findings of mean heart dose reduction in free-breathing IMRT compared to free-breathing VMAT [28]. This proved that the use of IMRT for its simplicity over VMAT was sufficient to provide the same dosimetric advantage for both 4D-CT-based free-breathing and DIBH RT.

V_50%_Lung dose among all eight planning methods was statistically insignificant. Based on the literature, Aznar et al. concluded that the lung exposure to BC RT varied substantially between different countries and regimens, so the radiation-related toxicity risk of lung cancer, pneumonitis, and lung fibrosis can also vary [30]. Using breathing adaptation, prone or lateral decubitus patient positioning technique can further minimize the irradiated lung region and extent [30].

Limitations of this study included the following: (1) 12 of the 15 patients were qualitatively chosen for DIBH treatment based on the heart location and the expected irradiated volume under standard breast RT. Therefore, the results of this study were likely biased toward DIBH being the ideal treatment technique. However, VMAT and IMRT under 4D-CT-based free-breathing conditions were still clinically feasible, which aligned with the published guidelines and are potential options for patients who are not compliant with breath-hold treatment, for situations where the distance between the heart and the chest wall is large, or in situations where centers are not able to offer DIBH; (2) the time duration per respiratory phase was not considered in 4D robust optimization dose calculation; (3) visualizing the LAD on both 4D-CT and DIBH CT datasets is difficult for optimal accuracy of the LAD delineation without the use of intravenous iodine contrast; (4) the time taken for contouring the LAD, LV, and DIBH in IMRT/VMAT was not considered in this study, in future studies, cardiac atlas and automation can be utilized to improve the treatment planning efficiency in sparing of the cardiac substructures.

This study tested for the statistical significance of various heart sparing RT techniques including 4D robust optimization and DIBH, which aimed to provide BC patients with the optimal treatment approach taking into account cardiac sparing, target coverage, and treatment complexity. In the future, clinical significance must be evaluated, including functional cardiac imaging and clinical outcomes, in order to establish a dose-response relationship that can be used to drive future dose optimization objectives (i.e., cardiac substructures). Currently, there is a minimal study (Beaton et al.) that investigates the correlation of cardiac substructure radiation dose toward a clinical endpoint in the BC population [10]. This information may help aid in the design of new patient-specific treatment strategies that aim to minimize inadvertent heart damage and provide better dose constraint consensus guidelines for better quality radiation treatment standards.

## Conclusions

This study demonstrated the clinical feasibility of free-breathing 4D-CT-based optimization in limiting radiation dose to the heart and its substructures with both IMRT/VMAT for an early stage left-sided BC patient cohort. 4D robust optimization cannot fully replace DIBH nor outperform standard 4D-CT-based IMRT/VMAT except in terms of minimizing the LAD dose. In comparison, both forward and inverse IMRT DIBH techniques were dosimetrically advantageous in heart sparing. This was compared to standard 4D-CT- and DIBH-based VMAT, 4D-CT robust optimization, and other free-breathing IMRT treatment techniques, given that the simplicity of IMRT in cardiac and substructure sparing outperformed VMAT technique. Despite the dosimetric advantage of DIBH with fixed-beam IMRT, all techniques had clinically acceptable plans according to published guidelines. Therefore, free-breathing 4D-CT-based techniques may be considered for patients who are not compliant with DIBH, where the heart and chest wall are far apart, or for centers where DIBH treatments are not available.
